# Analysis of strain distribution, migratory potential, and invasion history of fall armyworm populations in northern Sub-Saharan Africa

**DOI:** 10.1038/s41598-018-21954-1

**Published:** 2018-02-27

**Authors:** Rodney N. Nagoshi, Georg Goergen, Kodjo Agbeko Tounou, Komi Agboka, Djima Koffi, Robert L. Meagher

**Affiliations:** 1 Center for Medical, Agricultural and Veterinary Entomology, United States Department of Agriculture-Agricultural Research Service, Gainesville, Florida United States of America; 2 International Institute of Tropical Agriculture (IITA), Cotonou, Benin; 3 Ecole Supérieure d’Agronomie, Université de Lomé, Lomé, Togo; 4 Africa Regional Postgraduate Programme in Insect Science, University of Ghana, Accra, Ghana

## Abstract

Fall armyworm (*Spodoptera frugiperda* J.E. Smith) is a noctuid moth pest endemic throughout the Western Hemisphere that has recently become widespread in sub-Saharan Africa. There is a strong expectation of significant damage to African maize crop yield and a high likelihood of further dispersal, putting the rest of the Eastern Hemisphere at risk. Specimens from multiple locations in six countries spanning the northern portion of the infested region were analyzed for genetic markers. The similarity of haplotypes between the African collections was consistent with a common origin, but significant differences in the relative frequency of the haplotypes indicated limitations in migration. The mitochondrial marker frequently used to identify two host strains appears to be compromised, making uncertain previous reports that both strains are present in Africa. This more extensive study confirmed initial indications based on Togo populations that Florida and the Greater Antilles are the likely source of at least a subset of the African infestation and further suggest an entry point in western Africa. The origin of a second subgroup is less clear as it was rarely found in the collections and has a haplotype that has not yet been observed in the Western Hemisphere.

## Introduction

The fall armyworm, *Spodoptera frugiperda* (J.E. Smith), is a noctuid moth indigenous to the Western Hemisphere where it has long been a major agricultural problem for both continents^1^. It is primarily a pest of maize but has a wide host range and is capable of feeding on over 80 plant species, periodically causing significant economic damage to rice, sorghum, millet, soybean, wheat, alfalfa, cotton, turf, and fodder crops.

Fall armyworm has recently become established in Africa, the first major introgression into the Eastern Hemisphere. The initial detection was on the island nation of São Tomé and Príncipe in April 2016, followed by outbreaks in the western African countries of Benin, Nigeria, Ghana, and Togo reported in June 2016^2,3^. As of October 2017, fall armyworm is present throughout most of sub-Saharan Africa^4^. The infested region is predominated by smallholder farming with maize typically the most important staple food and a major component of livestock feed^5^. The damage from fall armyworm to African maize production could total $3 billion (USD) over the next year^6^, with the potential for losses in other crops still to be determined.

The apparent rapid spread of the pest in Africa suggests robust migratory movements that would be consistent with fall armyworm behavior in North America. Infestations extend as far north as Canada, the result of annual long-distance migrations from wintering areas limited in North America to the southern portions of Florida and Texas, the Caribbean, and Mexico^1,7,8^. The timing of this behavior corresponds to the seasonal northward progression of warmer temperatures and maize planting that follows the winter season, and air transport systems favorable to northward long-distance flight^1^. Projections from a migration model based on these factors accurately predicted fall armyworm migration patterns as validated by genetic haplotype studies^9^. However, because climate, farming practices, and wind patterns are substantially different from North America, fall armyworm migratory behavior in Africa is uncertain, which is a considerable limitation to the development of regional and area-wide strategies for sustainable pest management.

Western Hemisphere fall armyworm consists of two subpopulations that are morphologically indistinguishable but differ in their host plant distribution and certain physiological features^10–12^. The rice-strain (R-strain) is most consistently found in millet and grass species associated with pasture habitats while the corn-strain (C-strain) prefers maize and sorghum^13–15^. The strains can only be identified using molecular markers whose association with specific host plants is generally consistent but not absolute^13,16,17^. It is not known whether this variability reflects inaccuracy of the markers or plasticity in strain behavior. Contributing to the uncertainty is evidence that the strains are capable of cross-hybridization in the field, though at a significantly reduced frequency relative to within strain matings^18,19^. The behavior of these hybrids is not well understood, but they are preferentially found in maize habitats and so appear to retain a C-strain host bias^20^.

The C-strain can be subdivided into two geographically divided subgroups (FL-type and TX-type) on the basis of differences in the frequency of mitochondrial haplotypes^21,22^. The TX-type profile is found in most of the Western Hemisphere, with the FL-type limited to Florida and the Caribbean, as well as populations that migrate from Florida to annually infest the eastern coast of the U.S.^1,23–25^. The C-strain fall armyworm collected from the African country of Togo had a haplotype pattern most consistent with the FL-type^19^. Analogous haplotype differences for the R-strain have not yet been found, so it is unclear whether this group shows a similar geographical segregation.

In this paper we expand on the previous Togo study to include specimens from multiple locations in the island nations of São Tomé and Príncipe (Sao Tome) that lies approximately 300 km from the Africa mainland, Burundi, Democratic Republic of Congo (D.R. Congo), Tanzania, and Kenya (Table 1). Genetic analysis was performed to confirm the fall armyworm species identification, determine host strain identity, and identify polymorphisms that can distinguish between geographically separated African populations. Comparisons between the haplotypes found in the different African locations and with those prevalent in the Western Hemisphere were used to extrapolate likely migratory source locations and the magnitude of fall armyworm population movements. The implications of these results on the origins of the fall armyworm introduction into Africa, the behavior and presence of the host strains, and migration patterns are discussed.

**Table 1:** Source information for fall armyworm collections used in this study (DRC: Democratic Republic of Congo; Sao Tome: São Tomé and Príncipe).

Country	Region/Province	Year	Latitude	Longitude	n
Burundi	Cibitoke	2016	−2.8869	29.1248	11
Burundi	Bujumbura Mairie	2017	−3.3065	29.3251	12
Burundi	Bubanza	2017	−3.1393	29.2896	14
Burundi	Rumonge	2017	−3.9731	29.4382	12
DRC	Haut-Katanga	2017	Multiple sites		20
DRC	Sud-Ubangi	2017	Multiple sites		27
Kenya	Meru	2017	−0.0579	37.4681	10
Kenya	Nakuru	2017	−0.5214	36.0907	10
Kenya	Homa Bay	2017	−0.5046	34.4345	9
Kenya	Kirinyaga	2017	−0.3099	37.2696	6
Kenya	Kericho	2017	−0.1241	35.3361	10
Kenya	Narok	2017	−0.9776	35.0463	10
Sao Tome	Caue	2016	0.0351	6.5315	11
Sao Tome	Cantagalo	2016	0.2853	6.7162	11
Tanzania	Morogoro	2017	Multiple sites		69
Togo	Maritime	2016	Multiple sites		11
Togo	Plateaux	2016	6.9889	0.6537	9
Published Collections			Reference		
Togo			Nagoshi *et al*.^25^		
Argentina, Bolivia, Brazil, Peru, Puerto Rico, Dominican Republic			Nagoshi *et al*.^22^		
Florida, Texas			Nagoshi *et al*.^21,23^		

## Results

**Figure 1:**
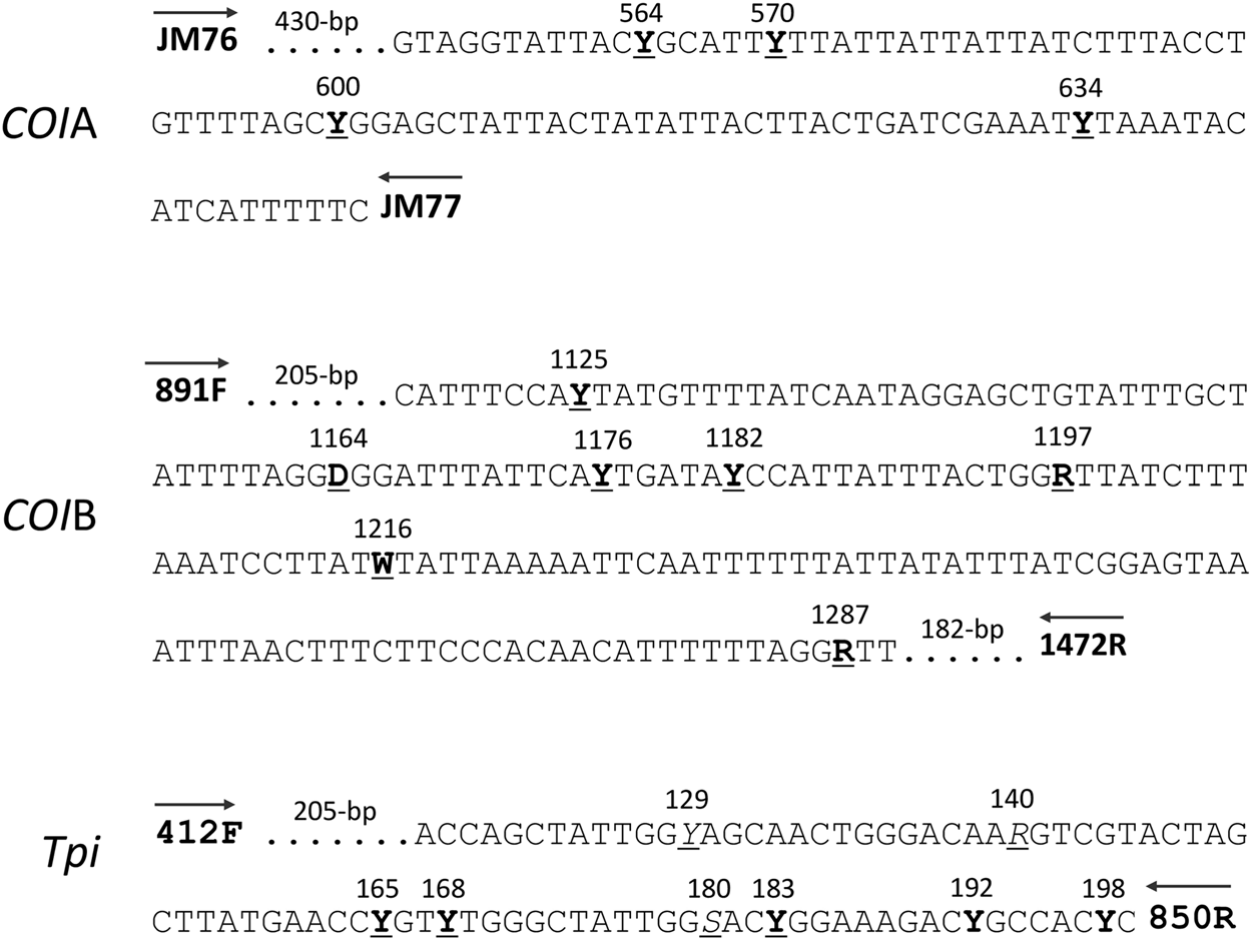
DNA segments from the *COI* and *Tpi* genes analyzed in this study are described showing sites with base substitutions with the observed alternatives indicated by IUPAC symbols. Sites in bold and underlined show strain-specific variation in Western Hemisphere populations. Three sites in the *Tpi* fragment showed additional variation in the African populations (underlined and italicized) and two sites had variations that were not strain-specific. Sites 165, 168, and 183 had been previously described as 352, 355, and 370, respectively^20,29^. Primers used for PCR amplification and DNA sequencing are displayed at the ends of each DNA segment.

Fall armyworm identified by morphological criteria were analyzed by sequence analysis of segments from the presumptive coding region of the mitochondrial *Cytochrome Oxidase Subunit I* (*COI*) gene and the sex-linked *Triosephosphate isomerase* (*Tpi*) gene. All sequence variations observed were single base substitutions that did not alter the predicted amino acid sequence (Fig. 1).

### Little *COI* variation in Africa

**Figure 2:**
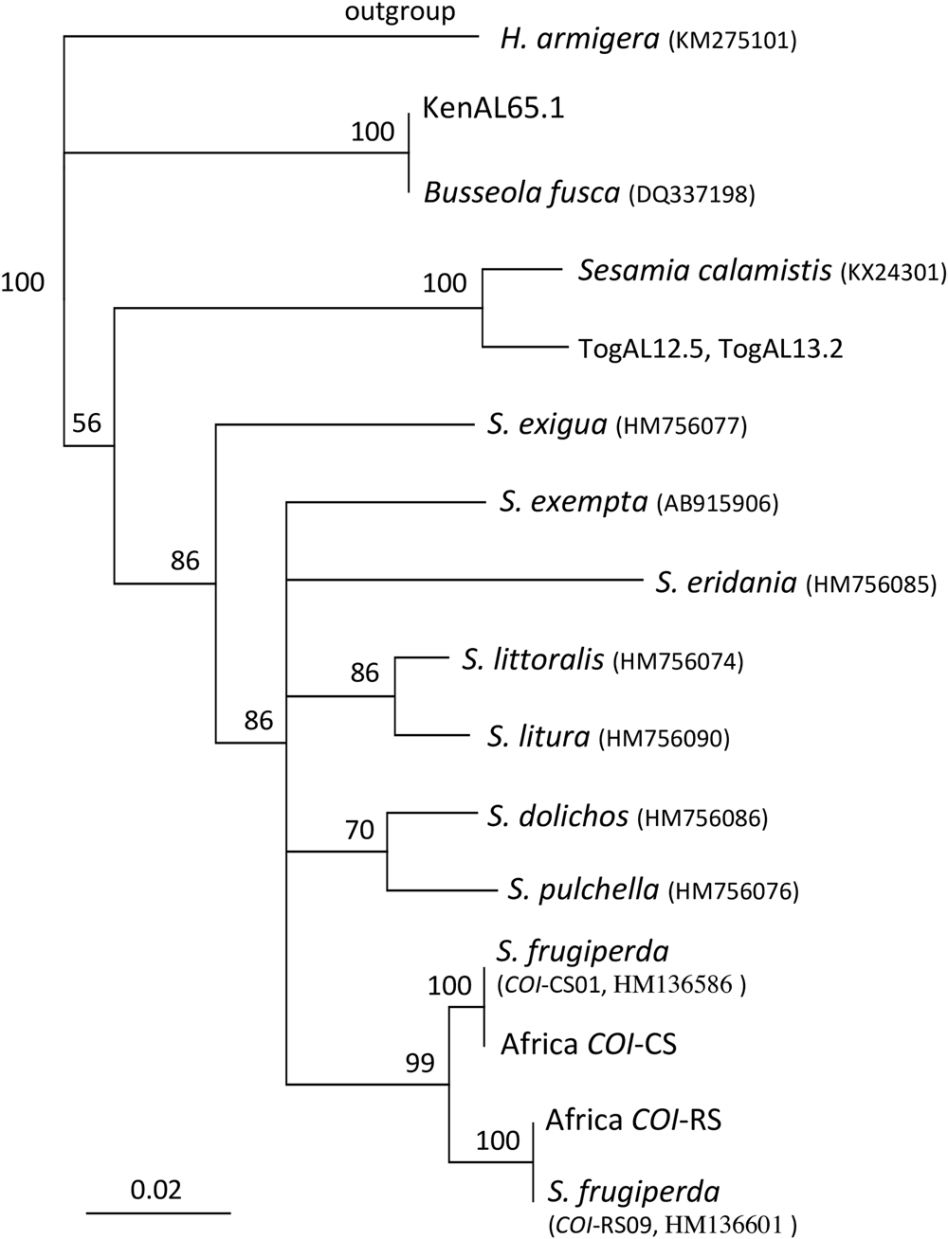
Strict consensus phylogenetic tree derived from neighbor- joining analysis comparing the two African *COI* haplotypes (*COI*) with those from fall armyworm host strains and related *Spodoptera* species^27^. *COI*-CS01 and *COI*-RS09 are fall armyworm haplotypes common to the Western Hemisphere fall armyworm. The analogous *COI* barcode segment from *Helicoverpa armigera* was use as the outgroup. The tree is based on Kimura-2-Parameter distances. Numbers at branch points indicate 2000X bootstrap values. Scale bar represents substitutions per site. GenBank accession numbers are provided for each species.

The CO1A segment was used to confirm species identification by comparisons with barcode sequences in GenBank. Of the 188 African specimens analyzed for CO1A, three displayed barcodes inconsistent with fall armyworm (Fig. 2). Two from Togo (TogAL12.5 and TogAL13.2) shared a haplotype most similar to *Sesamia calamistis* Hampson (Lepidoptera: Noctuidae) and one from Kenya (KenAL65.1) clustered with *Busseola fusca* Fuller (Lepidoptera: Noctuidae). Both species are native to Africa and are pests of maize and sorghum. The remaining specimens contained two CO1A haplotypes identical to that found in a previous study of Togo fall armyworms^26^. One sequence was identical to a variant of the C-strain group (*COI*-CS01) and the other to a R-strain haplotype (*COI*-RS09) that are both common in the Western Hemisphere^27^. The *COI* strain grouping determined by CO1A was confirmed by analysis of the downstream segment COIB. Again, only two haplotypes were observed, one for each strain. These differed at the same 12 sites that differentiate the consensus *COI*-CS and *COI*-RS haplotypes derived from Western Hemisphere populations (Table 2).

**Table 2:** Single nucleotide differences between the consensus *COI*-CS and *COI*-RS haplotypes based on Western Hemisphere (W.H.) fall armyworm for the COIA and COIB segments compared to that found in Africa (AFR). The numbers of each haplotype found in the Africa and Western Hemisphere collections from maize and sorghum habitats are listed.

Haplotype	COIA					COIB							Africa	W.H.
	489	564	570	600	634	1125	1164	1176	1182	1197	1216	1287		
W.H. *COI*-CS	T	T	C	C	T	T	R	T	C	G	T	R		427
W.H. *COI*-RS	C	C	T	T	C	C	T	C	T	A	A	A		272
AFR *COI*-CS	T	T	C	C	T	T	G	T	C	G	T	G	70	
AFR *COI*-RS	C	C	T	T	C	C	T	C	T	A	A	A	180	

### COIB haplotype suggests a Florida-Caribbean origin

**Figure 3:**
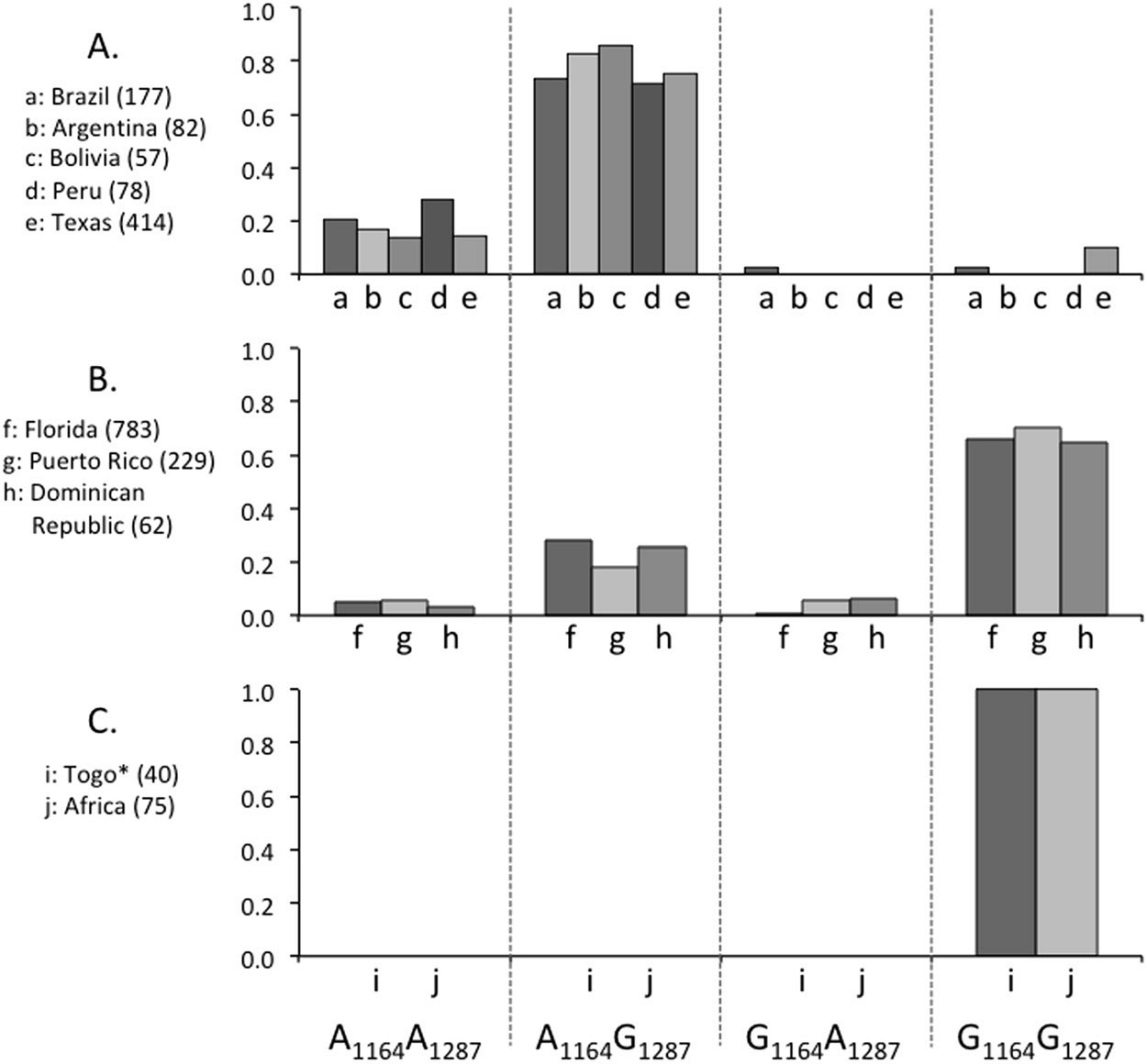
Frequency distributions of the COIB haplotype that differentiates geographical populations in the Western Hemisphere are described for Africa. The Y-axis indicates proportion of the population sampled. Numbers in parenthesis are total samples tested. Asterisk indicates data from previous study^26^.

Variants of two sites in the COIB segment, mCOI1164D and mCOI1287R, show geographical differences in frequency. In the Western Hemisphere *COI*-CS subgroup both sites vary by either an A or a G, producing the configurations of A_1164_A_1287_, A_1164_G_1287_, G_1164_A_1287_, and G_1164_G_1287_. The A_1164_G_1287_ subtype predominates in South America and Texas while G_1164_G_1287_ is the majority form in Florida and the Greater Antilles^22,24^ (Fig. 3A,B). In comparison, the 70 C-strain fall armyworm specimens characterized from Africa were all of the G_1164_G_1287_ haplotype, consistent with the results of 43 specimens previously examined from Togo^26^ (Fig. 3C).

### Disagreement between *COI* and *Tpi* markers

All specimens from Africa were collected from maize or sorghum, host plants associated with the C-strain population. Yet surprisingly the majority of specimens (72%, 180/250) carried the R-strain *COI*-RS haplotype rather than the expected *COI*-CS majority observed in the Western Hemisphere collections (Table 2). A subset of these was additionally tested for the *Tpi* strain markers, which gave a very different result. The C-strain *Tpi-*C haplotype as defined by C_183_ was found in 86% (203/243) of the African specimens with only three displaying the R-strain *Tpi-*R marker (Table 3A). These frequencies were comparable to that observed in the previous study of Togo fall armyworm and similar to that found in Western Hemisphere collections from analogous C-strain host plants (Table 3A).

**Table 3:** *Tpi* haplotypes observed in Africa (AFR) are defined based on differential base substitutions and their frequencies are compared with that observed in the Western Hemisphere (W.H.). Variants shown to be strain-specific in W.H. populations are italicized and in bold. Togo data from Nagoshi *et al*.^26^.

A. Tpi categories	Tpi SNPs										
	129	144	*165*	*168*	180	*183*	192	198	Togo	AFR	W.H.
a. *Tpi-*C						C			58	203	435
b. *Tpi-*R						T			2	3	174
c. *Tpi-*H (a/b)						Y			2	37	90
B. *Tpi*-C category											
d. *Tpi-*Ca1	C	G	C	T	C	C	C	C	0.37	0.60	0.33
e. *Tpi*-Ca2	C	G	C	T	C	C	T	T	0.27	0.07	0.22
f. *Tpi*-CaH (d/e)	C	G	C	T	C	C	Y	Y	0.37	0.33	0.37
other									0.00	0.00	0.08
C. *Tpi-*R category											
g. *Tpi*-Ra1	T	A	C	C	G	T	C	C	1.00	1.00	0.00
W.H. Variants	C	G	T	C	C	T			0.00	0.00	0.79
	C	G	T	C	T	T			0.00	0.00	0.08
	C	G	C	T	C	T			0.00	0.00	0.04
	C	G	A	C	C	T			0.00	0.00	0.03
	C	G	G	C	C	T			0.00	0.00	0.03
	A	G	T	C	C	T			0.00	0.00	0.01
	C	A	T	C	C	T			0.00	0.00	0.01
	C	G	C	C	T	T			0.00	0.00	0.01
	C	G	G	C	T	T			0.00	0.00	0.01
D. *Tpi-*h category											
TpiHafrCC (d/g)	Y	R	C	Y	S	Y	C	C	0.09	0.59	0.00
TpiHafrYY (e/g)	Y	R	C	Y	S	Y	Y	Y	0.91	0.41	0.00

### Geographical differences in the distribution of *COI* and *Tpi* haplotypes

**Figure 4:**
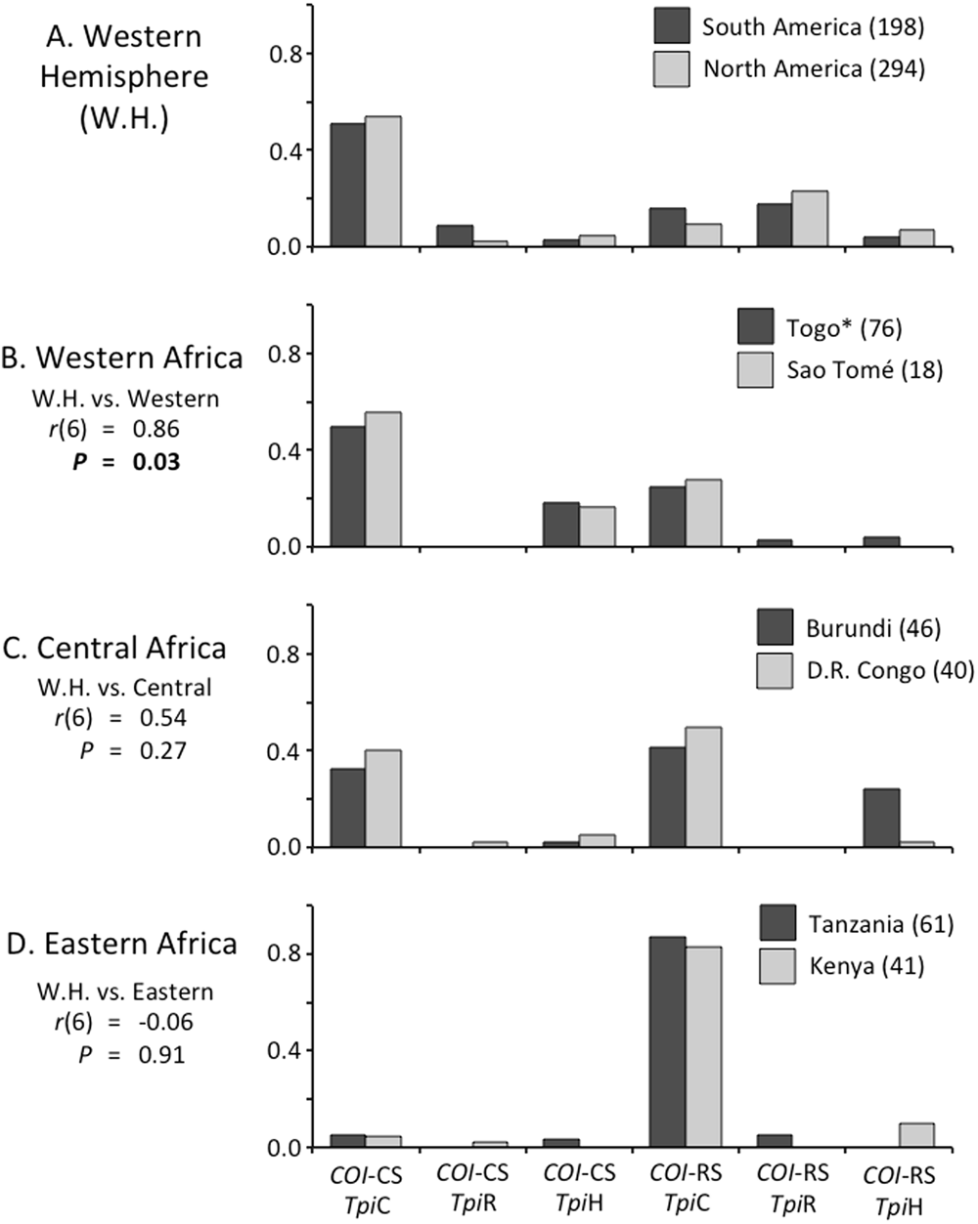
Frequencies of *COI* and *Tpi* haplotype combinations for different regions in Africa are described and compared to those found in fall armyworm from the Americas. Haplotypes are configurations represent those found in all collections and can be categorized as concordant, *COI*-CS *Tpi*-C and *COI*-RS *Tpi*-R; discordant, *COI*-CS *Tpi*-R and *COI*-RS *Tpi*-C, and hybrid, *COI*-CS *Tpi*-h, and *COI*-RS *Tpi*-h. The Western Hemisphere (W.H.) graph includes data from South America (Argentina, Brazil, Bolivia) and North America (Florida, Texas). Statistical metrics are from Pearson correlation tests comparing the pooled W.H. profile with the pooled frequency profiles from each region. The value for Togo (*) combines new data with that previously published^26^. Numbers after locations denote total samples analyzed.

The sex-linked *Tpi* gene segregates independently from the maternally-inherited mitochondrial *COI* gene. Despite this, Western Hemisphere fall armyworm populations consistently displayed high levels of concordance between the markers, i.e., marker configurations of *CO1*-CS *Tpi-*C and *COI*-RS *Tpi-*R, which suggests strong biases for mating within rather than between strains (Fig. 4A). In pooled collections from C-strain host plants in the Americas, 51% were *CO1*-CS Tpi-*C* and 26% *COI*-RS *Tpi-*R, giving a concordance frequency of 77%. This Western Hemisphere profile showed a significant positive correlation with that found for the two western Africa nations of Togo and Sao Tome, where the pooled collections also had a majority (53%) concordance (Fig. 4B). In contrast, the Western Hemisphere profile showed no significant correlation with profiles from the central or eastern collection sites (Fig. 4C,D). The central collections from Burundi and D. R. Congo appeared to give an intermediate pattern with roughly equal amounts of the concordant and discordant types (Fig. 4C), while the most eastern sites (Tanzania, Kenya) were dominated by the discordant *COI*-RS *Tpi-*C configuration (Fig. 4D).

**Figure 5:**
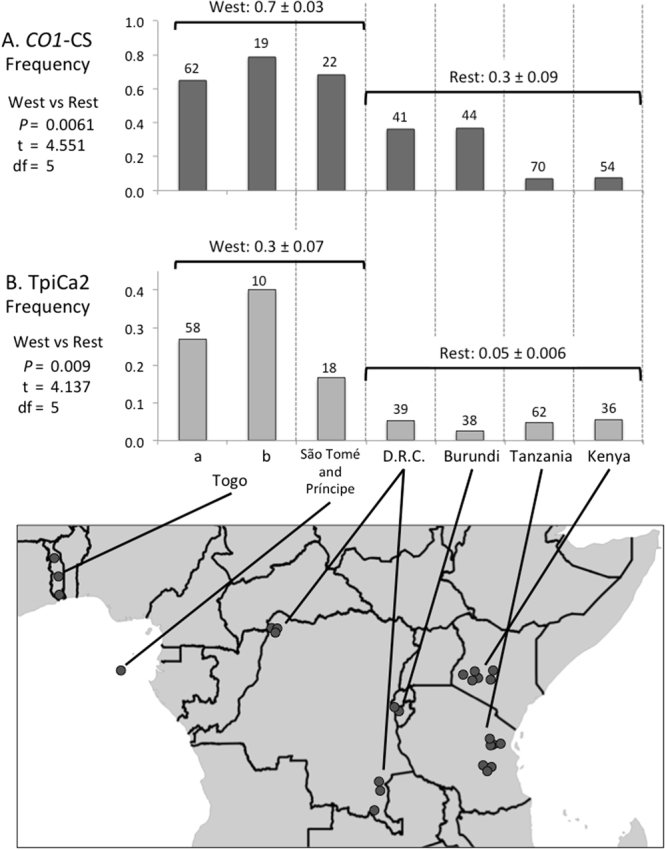
Frequencies of *COI*-CS and *Tpi*-Ca2 haplotype frequencies for different regions are indicated on a map of Africa. Togo (**B**) data are from previous study^26^, Togo (**B**) data from this study. Numbers above columns indicate total specimens for each collection. Means for groupings are indicated above horizontal line with Standard Error of the Mean. Statistical metrics are from two-tailed parametric *t*-test comparing the two means (West and Rest) for each haplotype. Circles on map approximate locations of sample collections. The map was created using Quantum Geographic Information System version 2.18.2 (QGIS Development Team (2016). QGIS Geographic Information System. Open Source Geospatial Foundation Project. http://qgis.osgeo.org).

The distribution of the *COI*-CS haplotype showed substantial differences between collections that also appeared to be aligned along an east-west axis (Fig. 5A). The *COI*-CS group was the majority in collections from the two most western African nations tested (Togo, Sao Tome) and progressively declined eastward, with the two most eastern locations (Kenya and Tanzania) showing a frequency of less than 10%. The mean frequency for Togo and Sao Tome was significantly different from that of the more eastern collections.

Two additional sites in the *Tpi* exon were previously shown to be highly variable in the Western Hemisphere but with much reduced strain specificity^19^. Sites gTpi192Y and gTpi198Y were similarly polymorphic in the Africa collection, subdividing the *Tpi-*C group into two haplotypes designated *Tpi-*Ca1 (with C_192_C_198_) and *Tpi-*Ca2 (T_192_T_198_), as well as a third group, *Tpi-*CaH (Y_192_Y_198_) whose DNA chromatograph pattern was consistent with a *Tpi-*Ca1/*Tpi-*Ca2 heterozygote (Table 2B). All three of these haplotype groups were also found in the Western Hemisphere at similar proportions. Once again there is evidence of east-west differences in frequency, with populations from Togo and Sao Tome showing the highest *Tpi*-Ca2 frequencies that were significantly different from the other collections (Fig. 5B).

### The African *Tpi*-R haplotype appears unique

Comparisons between *Tpi-*Ca1 and *Tpi-*Ca2 with the African *Tpi-*R variant (designated *Tpi-*Ra1) identified five differing sites in addition to the strain diagnostic gTpi183Y. Two sites, gTpi165Y and gTpi168Y, exhibit strain-specific variation in Western Hemisphere populations with >80% of *Tpi*-R specimens showing a different nucleotide than found in *Tpi*-C (Table 3BC). In contrast, only gTpi168Y differed between the African *Tpi-*C and *Tpi-*R variants (Table 3BC). In addition, *Tpi-*Ra1 differed from both *Tpi*-Ca1 and *Tpi*-Ca2 at three sites (gTpi129Y, gTpi144R, and gTpi180S) that are not strain-specific and are infrequently variable in the Western Hemisphere collections. To date the African *Tpi-*Ra1 haplotype has not been found in a survey of 699 specimens from the Western Hemisphere (Table 3C).

The DNA sequence chromatographs for the *Tpi-*h group showed overlapping peaks at each of the sites that differed between the two African strain haplotypes (Table 3D). This pattern is consistent with heterozygosity between *Tpi-*Ra1 and either *Tpi-*Ca1 or *Tpi-*Ca2, indicating that the two African host strains as defined by *Tpi* can productively hybridize.

## Discussion

In this and a previous study^26^, approximately 300 specimens from six African nations have been genetically characterized for markers in the *COI* and *Tpi* genes. The results show very little genetic variability in the African fall armyworm population with only two *COI* and three *Tpi* haplotypes found. Both *COI* variants and the two *Tpi-*Ca haplotypes were observed in all Africa locations and are common forms in the Western Hemisphere. In contrast, the African *Tpi-*R variant, *Tpi-*Ra1, is so far unique to Africa (Table 3). Only four *Tpi-*Ra1 specimens were found, two from Togo (both *COI*-RS), one from D. R. Congo (*COI*-CS), and one from Kenya (*COI*-RS), while the putative *Tpi-*Ra1/*Tpi-*CSa heterozygotes (the *Tpi*-h category) were present in all locations. Therefore, while the *Tpi-*Ra1 haplotype is rare in the collections from maize and sorghum, it is widely disseminated. These results in conjunction with the first detection of African fall armyworm infestations in 2016–2017^26^ are consistent with a recent introduction of a small invasive C-strain population that migrated rapidly throughout the northern Sub-Saharan region of the African continent. The history of the *Tpi*-Ra1 subgroup is less clear, as too few have been collected to test for regional differences and its presence in the Western Hemisphere has yet to be demonstrated.

While the fall armyworm in the surveyed portion of Africa shared a common source, geographical differences in haplotype frequencies suggest that long distance movements between distant locations were not of sufficient magnitude or consistency to homogenize geographically distant populations. Geographical structure is indicated by three haplotype distributions, each consistent with dispersion along an east-west axis (Figs 4 and 5). If these differences persist over time it would indicate that annual migrations in Africa tend to be more regional than continental, with the described marker combinations providing a means to define migratory limits and the area sharing a common population.

Current data are too preliminary to make definitive conclusions about either the Western Hemisphere source(s) of the invasive population or their site(s) of introduction into Africa. Multiyear data are needed as well as surveys from southern Africa. Nevertheless, the results provide some preliminary indications. The distribution of concordant and discordant *COI* and *Tpi* marker combinations vary considerably across Africa, with the frequency profile of the Western Hemisphere positively correlating with that of the most western collection sites (Togo and Sao Tome) but not to the other more eastern locations (Fig. 4). This finding is most consistent with fall armyworm from the Western Hemisphere first entering through western Africa.

There is also evidence that identify the likely Western Hemisphere source of the Africa fall armyworm. All 108 of the *CO1*-CS fall armyworm from Africa analyzed at COIB have the G_1164_G_1287_ SNP configuration. This is the predominant *CO1*-CS variant found in the Greater Antilles and Florida, with the latter being the source of migratory infestations in the eastern United States, but is rare in South America and relatively infrequent in Texas and the central regions of the United States. Therefore, we believe that the most likely source of the surveyed *COI*-CS fall armyworm in at least northern Sub-Saharan Africa are populations that normally winter in the Caribbean or Florida. This was first indicated by our earlier genetic survey of Togo^26^, with the additional collections confirming that finding and suggestive of a single introduction for the northern Sub-Saharan region. If multiple introductions occurred, it appears they all originated from the same Western Hemisphere source.

The origin of the *Tpi-*Ra1 haplotype is not clear and its apparent scarcity in the Western Hemisphere is surprising. The few *Tpi*-Ra1 specimens found carry the same two *CO1* haplotypes as the African *CO1*-CS group, suggesting that both originated from a common source. Therefore, we believe the most parsimonious explanation is a single introduction of both strains into Africa with a subset happening to carry a rare *Tpi* haplotype variant. However, we cannot completely discount the possibility that the *Tpi-*Ra1 subgroup may not be present in the Western Hemisphere and so has a different origin, perhaps even representing a fall armyworm population native to Africa. Furthermore, it is not clear whether the *Tpi-*Ra1 group is simply a variant of the African C-strain population (*i.e*., the gTpi183Y site is not strain-specific in Africa) or is representative of a more abundant R-strain that is present in habitats not yet surveyed. Characterization of additional *Tpi-*R specimens from Africa and surveys of different habitats should clarify this issue.

This and other studies have shown that the *COI*-RS haplotype is present throughout Africa and is the predominant group in some locations^2,3,26^. However, two observations indicate that *COI*-RS is not behaving as an accurate marker of the R-strain. The first is its high frequency in collections from traditional C-strain host plants (maize, sorghum), particularly in the central and eastern collection sites (Fig. 5A). While such events have been reported in the Western Hemisphere, they are not the norm^14,17,28^. The second is the predominance of the discordant configuration in parts of Africa, indicating dissociation between the *COI* and *Tpi* strain haplotypes (Fig. 4C,D). The reason for this loss of *COI* strain specificity can only be speculated, but could result from interstrain hybridization producing discordant configurations that by chance became predominant. Overall, we believe the data to date leaves uncertain whether the R-strain is present in Africa, particularly since the collections were limited to host plants preferred by the C-strain and so may not be representative of the total African population.

In conclusion, genetic characterization of portions of the *COI* and *Tpi* genes demonstrate a common origin for the recent infestation of fall armyworm over the northern portion of Sub-Saharan Africa and provide preliminary indications of where in Africa fall armyworm was first introduced and the likely Western Hemisphere source population. Haplotype distribution profiles identify differences between geographical populations that have the potential for defining fall armyworm migration patterns. This will have important ramifications for the design of area-wide strategies to mitigate infestations.

## Methods

### Specimen Collections and DNA preparation

Specimens were obtained as larvae from corn (maize) or sorghum plants at various locations in northern Sub-Saharan Africa at various times in 2016 (Table 1). Specimens were stored either air-dried or in ethanol at room temperature. A portion of each specimen was excised and homogenized in a 5-ml Dounce homogenizer (Thermo Fisher Scientific, Waltham, MA, USA) in 800 µl Genomic Lysis buffer (Zymo Research, Orange, CA, USA) and incubated at 55 °C for 5–30 min. Debris was removed by centrifugation at 10,000 rpm for 5 min. The supernatant was transferred to a Zymo-Spin III column (Zymo Research, Orange, CA, USA) and processed according to manufacturer’s instructions. The DNA preparation was increased to a final volume of 100 µl with distilled water. Genomic DNA preparations of fall armyworm samples from previous studies were stored at −20 °C.

### Genetic markers and nomenclature

The genetic markers are all single nucleotide substitutions. Sites in the *COI* gene are designated by an “m” (mitochondria) while *Tpi* sites are designated “g” (genomic). This is followed by the gene name, number of base pairs from the predicted translational start site (*COI*) or 5′ start of exon (*Tpi*), and the nucleotides observed using IUPAC convention (R: A or G, Y: C or T, W: A or T, K: G or T, S: C or G, D: A or G or T). Sites gTpi165Y, gTpi168Y, and gTpi183Y were previously described as 352, 355, and 370^20,29^.

### Characterization of the *CO1* and *Tpi* gene segments

The *COI* markers are from the maternally inherited mitochondrial genome. Two adjacent segments of *CO1* were analyzed by DNA sequencing. The *COI*A segment was amplified by the *COI* primers *JM76* and *JM77* and used to confirm species identity and to determine the fall armyworm *COI* strain haplotypes *COI*-CS (C-strain) and *CO1*-RS (R-strain)^30^. The DNA sequences of the fall armyworm host strains and other *Spodoptera* species were previously described and available in GenBank^27^. DNA alignments and consensus building were performed using MUSCLE (multiple sequence comparison by log-expectation), a public domain multiple alignment software incorporated into the Geneious Pro 10.1.2 program (Biomatters, New Zealand, http://www.geneious.com)^31^. Phylogenetic trees were graphically displayed in a neighbor-joining (NJ) tree analysis also included in the Geneious Pro 10.1.2 program^32^. The adjacent COIB segment was amplified by *CO1* primers 891 F and 1472 R and used to confirm host strain identity and determine the region-specific haplotypes^24^ (Fig. 1).

Variants in the *Triosephosphate isomerase* gene (*Tpi*) can also be used to identify host strain identity with results generally comparable with the *CO1* marker [30, 31]. The gTpi183Y site is on the fourth exon of the predicted *Tpi* coding region and was PCR amplified using the *Tpi* primers 412 F and 850 R (Fig. 1). The C-strain allele (*Tpi*-C) is indicated by a C_183_ and the R-strain (*Tpi*-R) by T_183_^20^. The *Tpi* gene is located on the *Z* sex chromosome that is present in one copy in females and two copies in males. Since males can be heterozygous for *Tpi*, there is the potential for the simultaneous display of both alternative nucleotides at *Tpi*_183_ (denoted as *Tpi-*h), which would be indicated by an overlapping C and T DNA sequence chromatograph^20^.

PCR amplification for all segments was performed in a 30-µl reaction mix containing 3 µl 10X manufacturer’s reaction buffer, 1 µl 10 mM dNTP, 0.5 µl 20-µM primer mix, 1 µl DNA template (between 0.05–0.5 µg), 0.5 unit Taq DNA polymerase (New England Biolabs, Beverly, MA). The thermocycling program was 94 °C (1 min), followed by 33 cycles of 92 °C (30 s), 56 °C (45 s), 72 °C (45 s), and a final segment of 72 °C for 3 min. Typically 96 PCR amplifications were performed at the same time using either 0.2-ml tube strips or 96 well microtiter plates. All primers were obtained from Integrated DNA Technologies (Coralville, IA). Amplification of the *CO1* barcode region was performed using primers *JM76* (5′-GAGCTGAATTAGGGACTCC-3′) and *JM77* (5′-ATCACCTCCACCTGCAGGATC-3′) to produce a 569-bp fragment (Fig. 1). Amplification of the *CO1* segment used to determine haplotypes used the primer pair *891 F* (5′-TACACGAGCATATTTTACATC-3′) and *1472 R* (5′-GCTGGTGGTAAATTTTGATATC-3′) to produce a 603-bp fragment. Amplification of the *Tpi* gene segment used the primers *412 F* (5′-CCGGACTGAAGGTTATCGCTTG-3′) and *850 R* (5′-AATTTTATTACCTGCTGTGG-3′) to produce a fragment containing most of the fourth exon with an approximate length of 199 bp.

For fragment isolations, 6 µl of 6X gel loading buffer was added to each amplification reaction and the entire sample run on a 1.8% agarose horizontal gel containing GelRed (Biotium, Hayward, CA) in 0.5X Tris-borate buffer (TBE, 45 mM Tris base, 45 mM boric acid, 1 mM EDTA pH 8.0). Fragments were visualized on a long-wave UV light box and manually cut out from the gel. Fragment isolation was performed using Zymo-Spin I columns (Zymo Research, Orange, CA) according to manufacturer’s instructions. The University of Florida Interdisciplinary Center for Biotechnology (Gainesville, FL) and Genewiz (South Plainfield, NJ) performed the DNA sequencing.

### Data Availability

All data generated or analysed during this study are included in this published article. Sequences deposited into GenBank include COIA haplotypes TogAL12.5 (MG603699), TogA13.2 (MG603700), KenAL65.1 (MG603701), and *Tpi* haplotypes *Tpi*-Ca1 (MG603702), *Tpi*-Ca2 (MG603703), and *Tpi*-Ra1 (MG603704).
